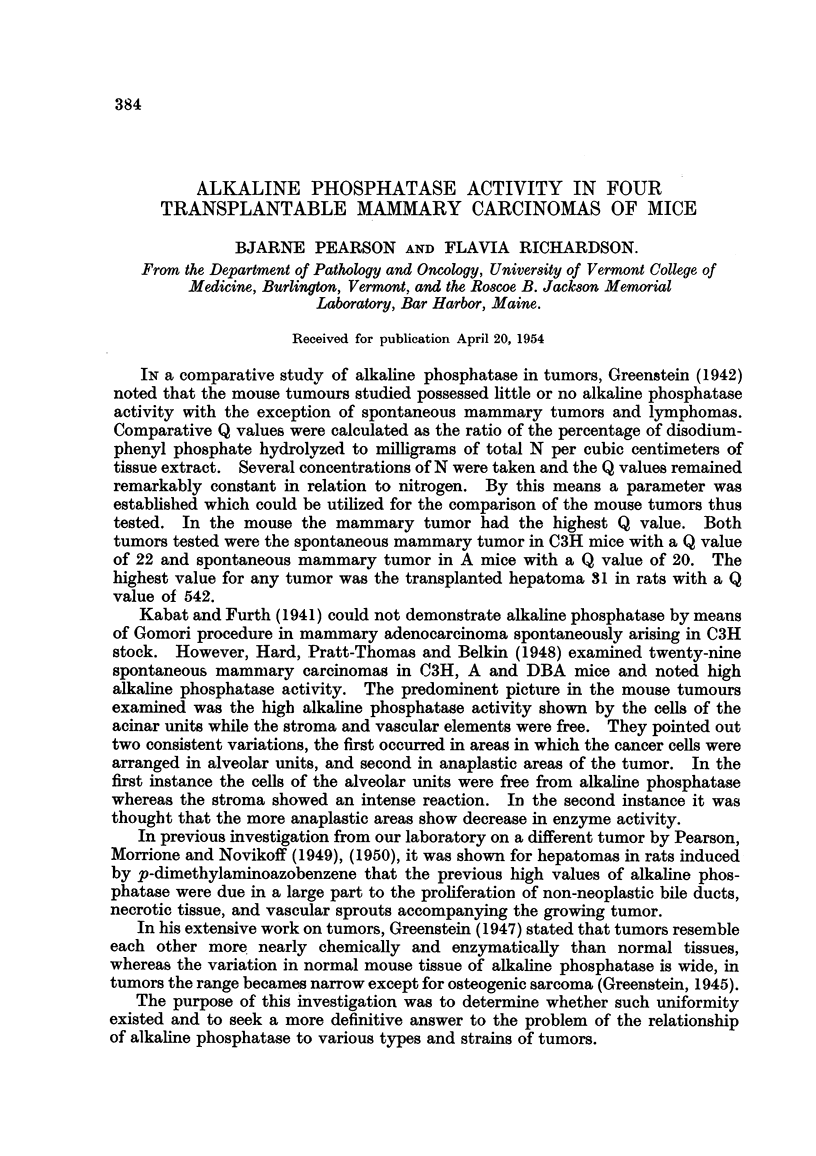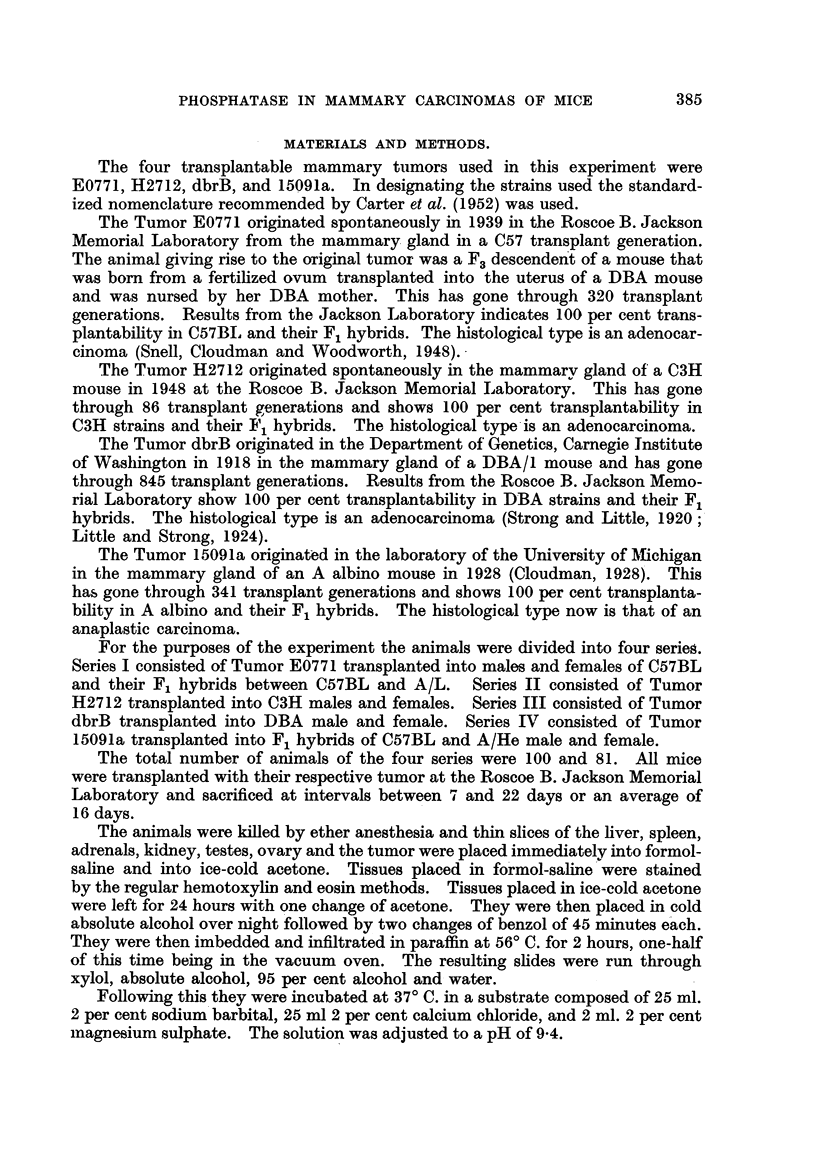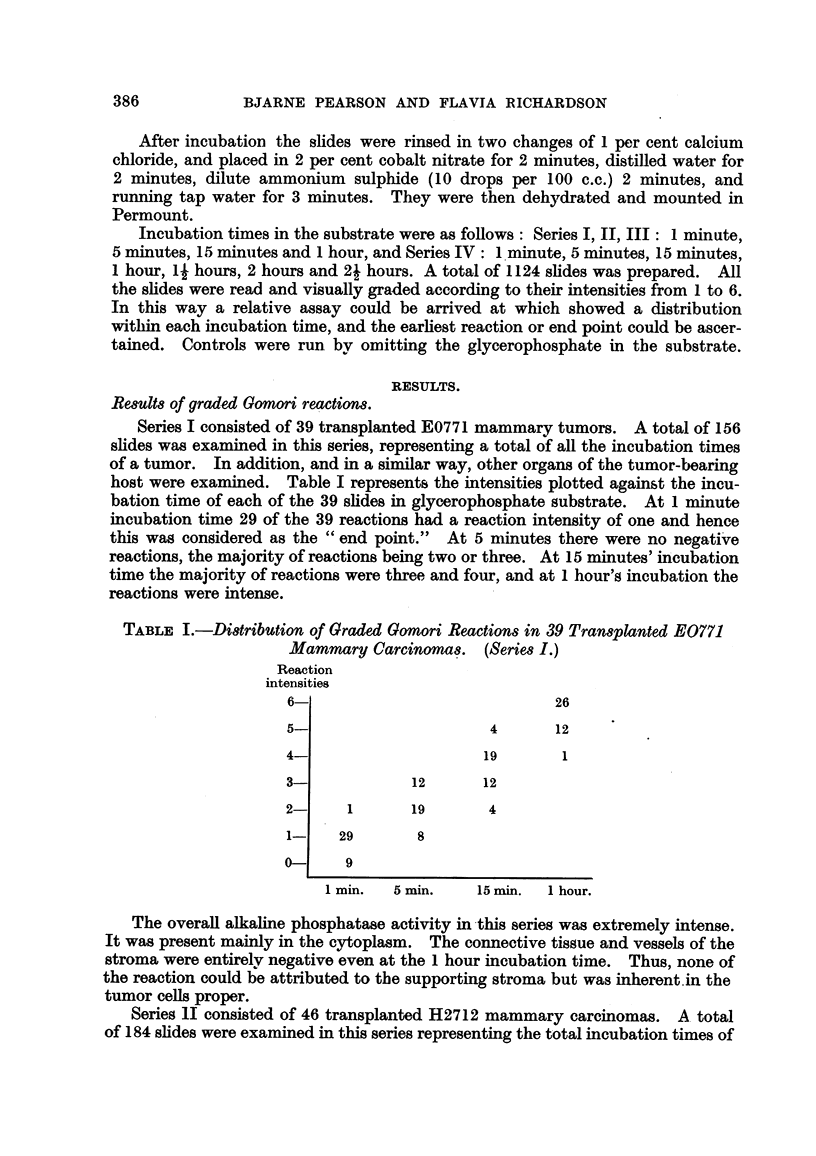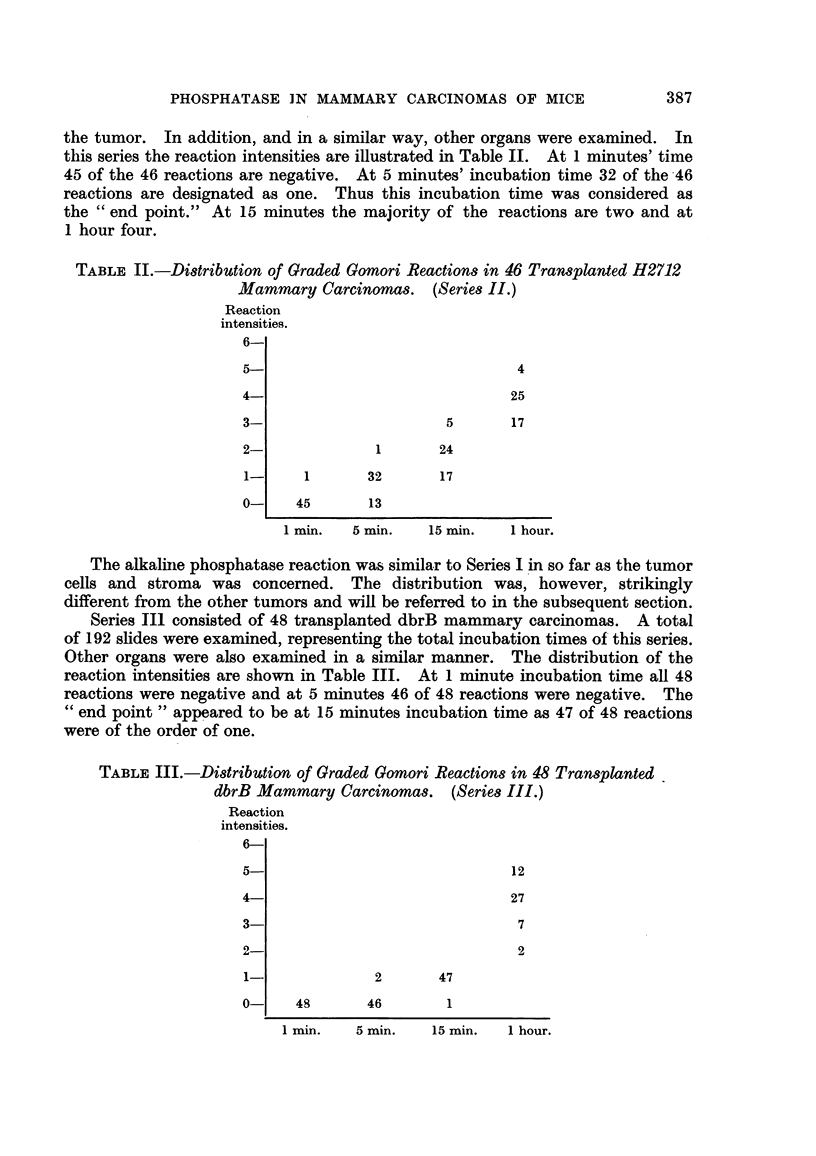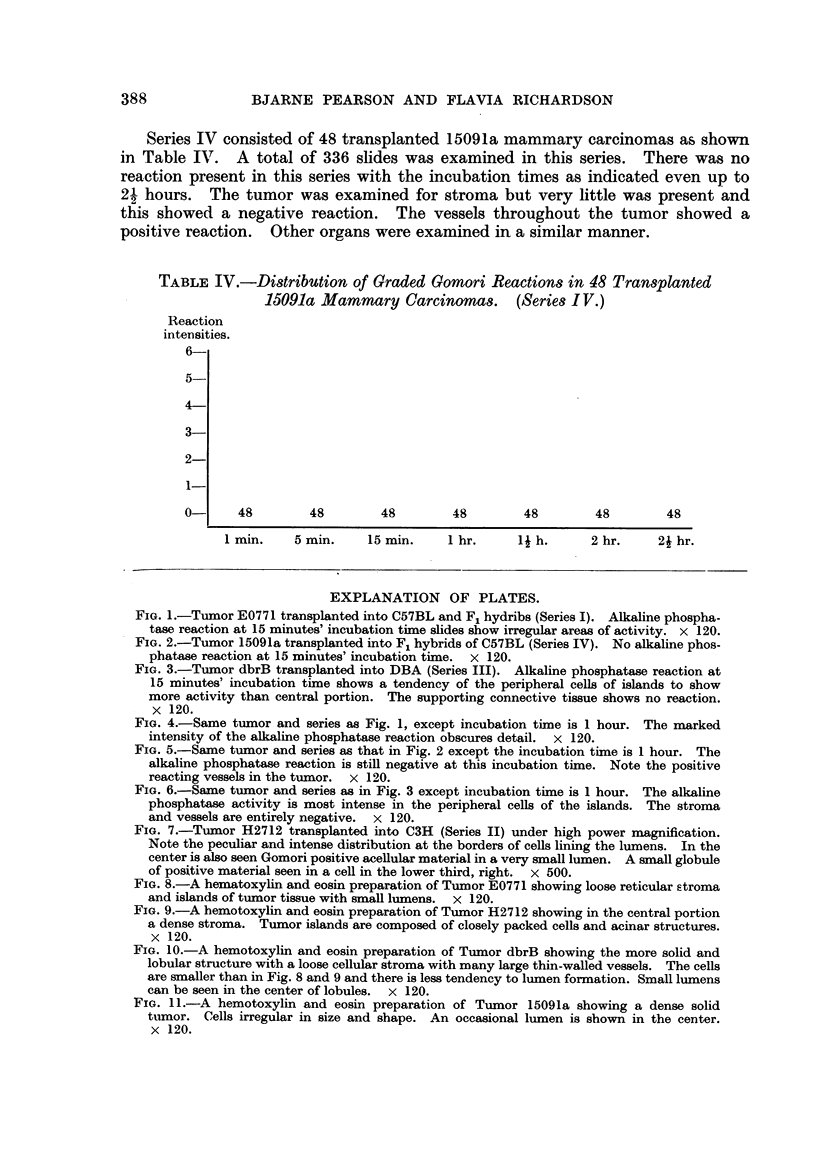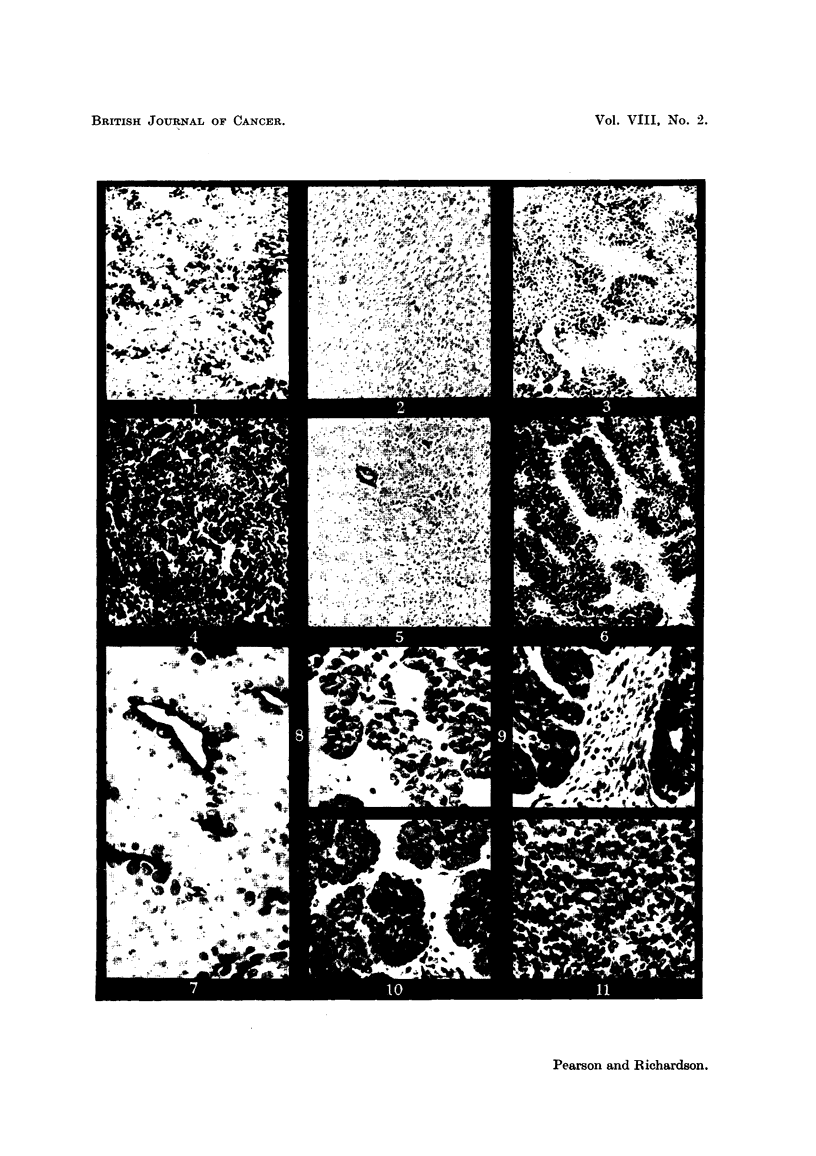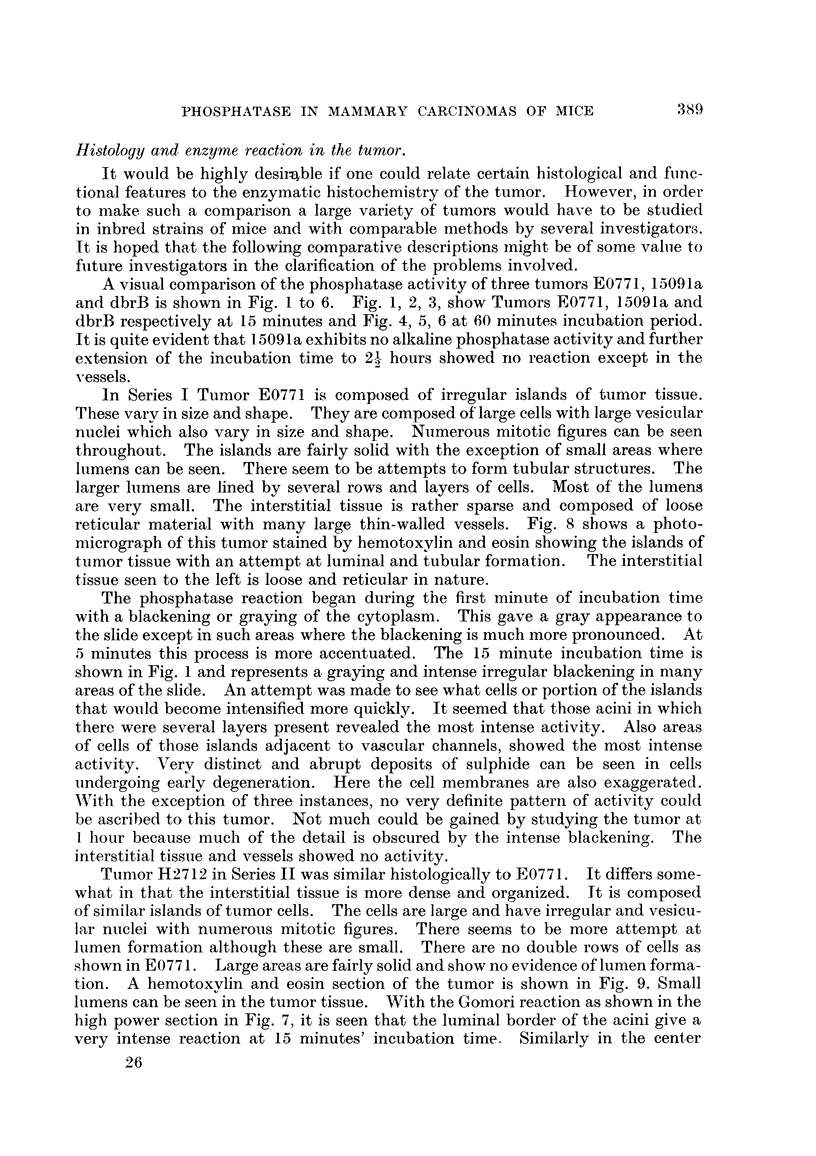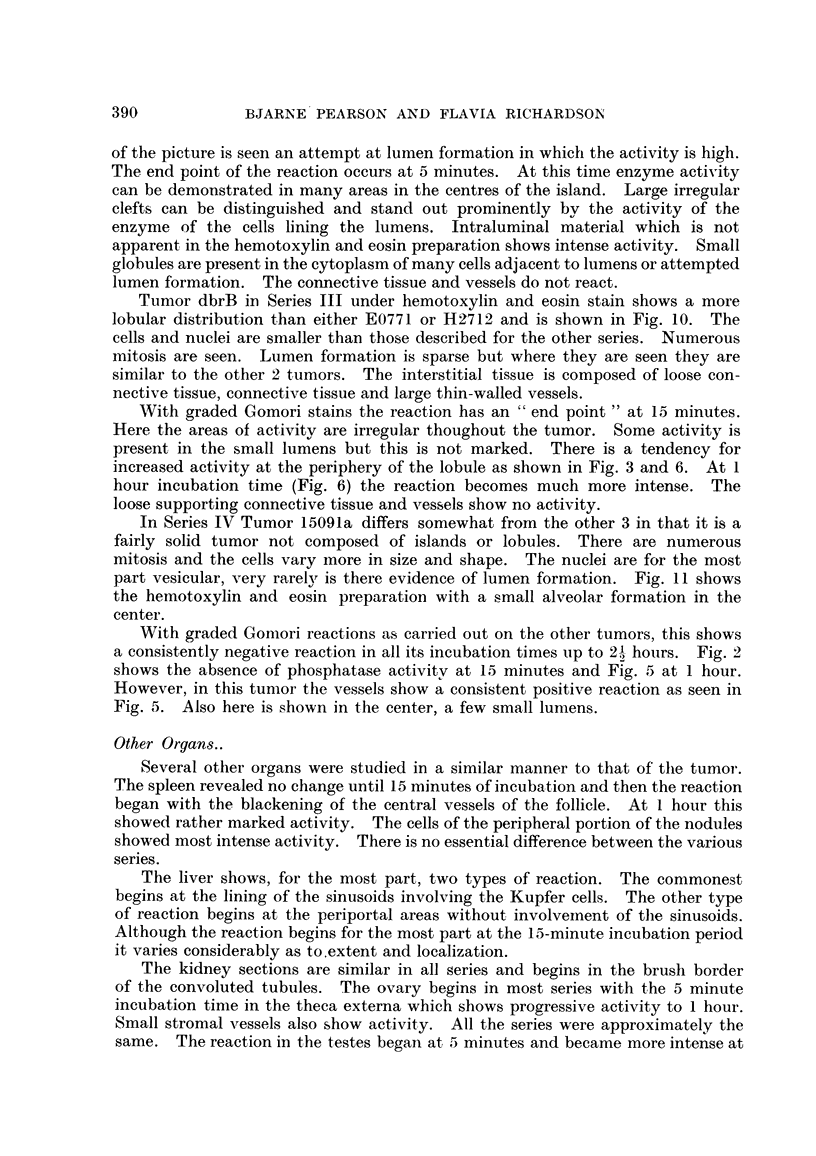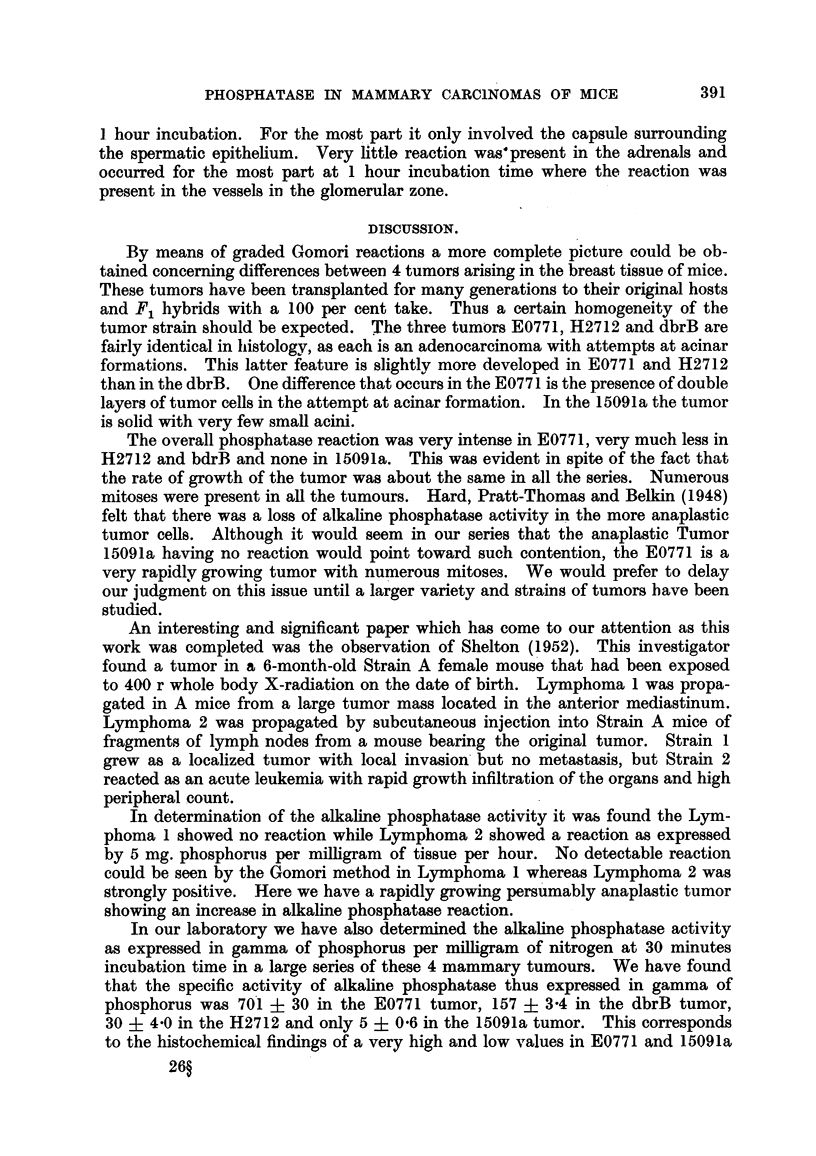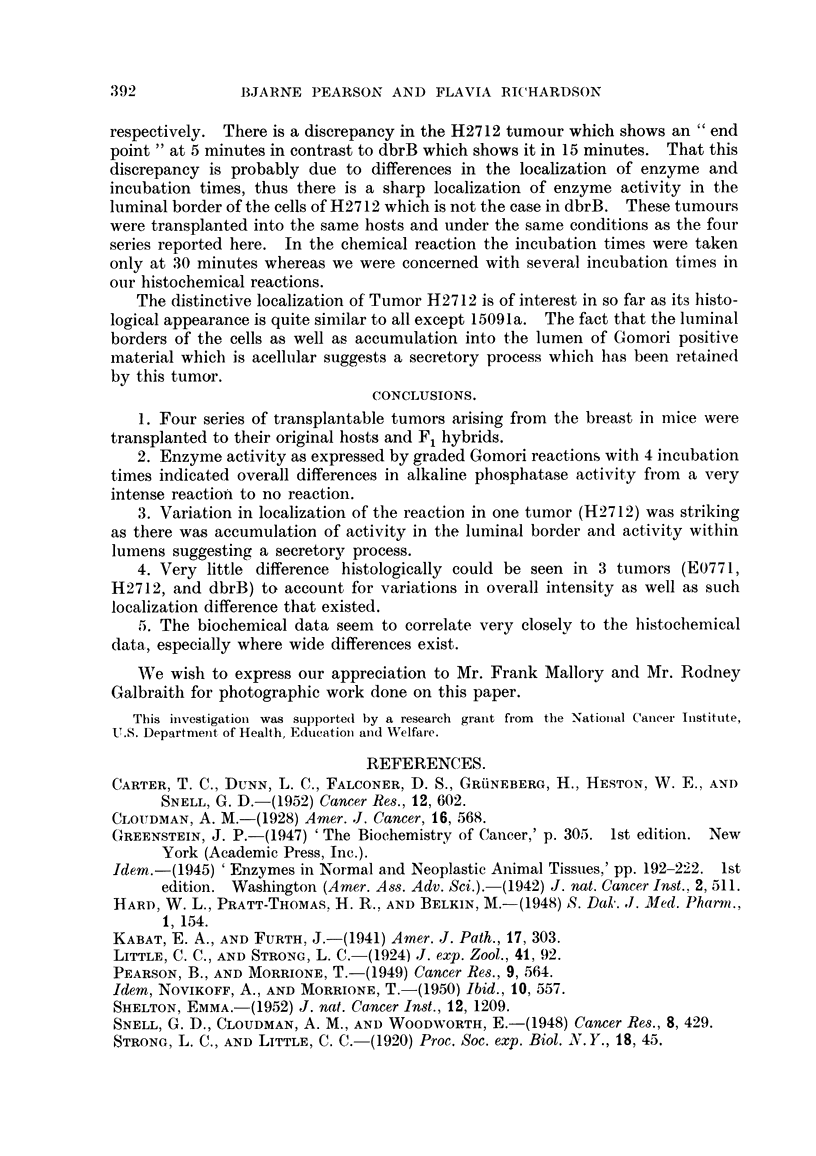# Alkaline Phosphatase Activity in Four Transplantable Mammary Carcinomas of Mice

**DOI:** 10.1038/bjc.1954.40

**Published:** 1954-06

**Authors:** Bjarne Pearson, Flavia Richardson

## Abstract

**Images:**


### ALKALINE PHOSPHATASE ACTIVITY IN FOUR TRANSPLANTABLE MAMMARY CARCINOMAS OF MICE

BJARNE PEARSON AND FLAVIA RICHARDSON.

*From the Department of Pathology and Oncology, University of Vermont College of Medicine, Burlington, Vermont, and the Roscoe B. Jackson Memorial Laboratory, Bar Harbor, Maine.*

Received for publication April 20, 1954

IN a comparative study of alkaline phosphatase in tumors, Greenstein (1942) noted that the mouse tumours studied possessed little or no alkaline phosphatase activity with the exception of spontaneous mammary tumors and lymphomas. Comparative Q values were calculated as the ratio of the percentage of disodium-phenyl phosphate hydrolyzed to milligrams of total N per cubic centimeters of tissue extract. Several concentrations of N were taken and the Q values remained remarkably constant in relation to nitrogen. By this means a parameter was established which could be utilized for the comparison of the mouse tumors thus tested. In the mouse the mammary tumor had the highest Q value. Both tumors tested were the spontaneous mammary tumor in C3H mice with a Q value of 22 and spontaneous mammary tumor in A mice with a Q value of 20. The highest value for any tumor was the transplanted hepatoma 31 in rats with a Q value of 542.

Kabat and Furth (1941) could not demonstrate alkaline phosphatase by means of Gomori procedure in mammary adenocarcinoma spontaneously arising in C3H stock. However, Hard, Pratt-Thomas and Belkin (1948) examined twenty-nine spontaneous mammary carcinomas in C3H, A and DBA mice and noted high alkaline phosphatase activity. The predominent picture in the mouse tumours examined was the high alkaline phosphatase activity shown by the cells of the acinar units while the stroma and vascular elements were free. They pointed out two consistent variations, the first occurred in areas in which the cancer cells were arranged in alveolar units, and second in anaplastic areas of the tumor. In the first instance the cells of the alveolar units were free from alkaline phosphatase whereas the stroma showed an intense reaction. In the second instance it was thought that the more anaplastic areas show decrease in enzyme activity.

In previous investigation from our laboratory on a different tumor by Pearson, Morrione and Novikoff (1949), (1950), it was shown for hepatomas in rats induced by *p*-dimethylaminoazobenzene that the previous high values of alkaline phosphatase were due in a large part to the proliferation of non-neoplastic bile ducts, necrotic tissue, and vascular sprouts accompanying the growing tumor.

In his extensive work on tumors, Greenstein (1947) stated that tumors resemble each other more nearly chemically and enzymatically than normal tissues, whereas the variation in normal mouse tissue of alkaline phosphatase is wide, in tumors the range becames narrow except for osteogenic sarcoma (Greenstein, 1945).

The purpose of this investigation was to determine whether such uniformity existed and to seek a more definitive answer to the problem of the relationship of alkaline phosphatase to various types and strains of tumors.

### MATERIALS AND METHODS.

The four transplantable mammary tumors used in this experiment were E0771, H2712, dbrB, and 15091a. In designating the strains used the standardized nomenclature recommended by Carter *et al.* (1952) was used.

The Tumor E0771 originated spontaneously in 1939 in the Roscoe B. Jackson Memorial Laboratory from the mammary gland in a C57 transplant generation. The animal giving rise to the original tumor was a $F_3$ descendent of a mouse that was born from a fertilized ovum transplanted into the uterus of a DBA mouse and was nursed by her DBA mother. This has gone through 320 transplant generations. Results from the Jackson Laboratory indicates 100 per cent transplantability in C57BL and their $F_1$ hybrids. The histological type is an adenocarcinoma (Snell, Cloudman and Woodworth, 1948).

The Tumor H2712 originated spontaneously in the mammary gland of a C3H mouse in 1948 at the Roscoe B. Jackson Memorial Laboratory. This has gone through 86 transplant generations and shows 100 per cent transplantability in C3H strains and their $F_1$ hybrids. The histological type is an adenocarcinoma.

The Tumor dbrB originated in the Department of Genetics, Carnegie Institute of Washington in 1918 in the mammary gland of a DBA/1 mouse and has gone through 845 transplant generations. Results from the Roscoe B. Jackson Memorial Laboratory show 100 per cent transplantability in DBA strains and their $F_1$ hybrids. The histological type is an adenocarcinoma (Strong and Little, 1920; Little and Strong, 1924).

The Tumor 15091a originated in the laboratory of the University of Michigan in the mammary gland of an A albino mouse in 1928 (Cloudman, 1928). This has gone through 341 transplant generations and shows 100 per cent transplantability in A albino and their $F_1$ hybrids. The histological type now is that of an anaplastic carcinoma.

For the purposes of the experiment the animals were divided into four series. Series I consisted of Tumor E0771 transplanted into males and females of C57BL and their $F_1$ hybrids between C57BL and A/L. Series II consisted of Tumor H2712 transplanted into C3H males and females. Series III consisted of Tumor dbrB transplanted into DBA male and female. Series IV consisted of Tumor 15091a transplanted into $F_1$ hybrids of C57BL and A/He male and female.

The total number of animals of the four series were 100 and 81. All mice were transplanted with their respective tumor at the Roscoe B. Jackson Memorial Laboratory and sacrificed at intervals between 7 and 22 days or an average of 16 days.

The animals were killed by ether anesthesia and thin slices of the liver, spleen, adrenals, kidney, testes, ovary and the tumor were placed immediately into formol-saline and into ice-cold acetone. Tissues placed in formol-saline were stained by the regular hemotoxylin and eosin methods. Tissues placed in ice-cold acetone were left for 24 hours with one change of acetone. They were then placed in cold absolute alcohol over night followed by two changes of benzol of 45 minutes each. They were then imbedded and infiltrated in paraffin at 56° C. for 2 hours, one-half of this time being in the vacuum oven. The resulting slides were run through xylol, absolute alcohol, 95 per cent alcohol and water.

Following this they were incubated at 37° C. in a substrate composed of 25 ml. 2 per cent sodium barbital, 25 ml 2 per cent calcium chloride, and 2 ml. 2 per cent magnesium sulphate. The solution was adjusted to a pH of 9·4.

After incubation the slides were rinsed in two changes of 1 per cent calcium chloride, and placed in 2 per cent cobalt nitrate for 2 minutes, distilled water for 2 minutes, dilute ammonium sulphide (10 drops per 100 c.c.) 2 minutes, and running tap water for 3 minutes. They were then dehydrated and mounted in Permount.

Incubation times in the substrate were as follows : Series I, II, III : 1 minute, 5 minutes, 15 minutes and 1 hour, and Series IV : 1 minute, 5 minutes, 15 minutes, 1 hour, 1½ hours, 2 hours and 2½ hours. A total of 1124 slides was prepared. All the slides were read and visually graded according to their intensities from 1 to 6. In this way a relative assay could be arrived at which showed a distribution within each incubation time, and the earliest reaction or end point could be ascertained. Controls were run by omitting the glycerophosphate in the substrate.

### RESULTS.

*Results of graded Gomori reactions.*

Series I consisted of 39 transplanted E0771 mammary tumors. A total of 156 slides was examined in this series, representing a total of all the incubation times of a tumor. In addition, and in a similar way, other organs of the tumor-bearing host were examined. Table I represents the intensities plotted against the incubation time of each of the 39 slides in glycerophosphate substrate. At 1 minute incubation time 29 of the 39 reactions had a reaction intensity of one and hence this was considered as the " end point." At 5 minutes there were no negative reactions, the majority of reactions being two or three. At 15 minutes' incubation time the majority of reactions were three and four, and at 1 hour's incubation the reactions were intense.

TABLE I.—*Distribution of Graded Gomori Reactions in 39 Transplanted E0771 Mammary Carcinomas. (Series I.)*

| Reaction intensities | 1 min. | 5 min. | 15 min. | 1 hour. |
|---|---|---|---|---|
| 6 | | | | 26 |
| 5 | | | 4 | 12 |
| 4 | | | 19 | 1 |
| 3 | | 12 | 12 | |
| 2 | 1 | 19 | 4 | |
| 1 | 29 | 8 | | |
| 0 | 9 | | | |

The overall alkaline phosphatase activity in this series was extremely intense. It was present mainly in the cytoplasm. The connective tissue and vessels of the stroma were entirely negative even at the 1 hour incubation time. Thus, none of the reaction could be attributed to the supporting stroma but was inherent in the tumor cells proper.

Series II consisted of 46 transplanted H2712 mammary carcinomas. A total of 184 slides were examined in this series representing the total incubation times of the tumor. In addition, and in a similar way, other organs were examined. In this series the reaction intensities are illustrated in Table II. At 1 minutes' time 45 of the 46 reactions are negative. At 5 minutes' incubation time 32 of the 46 reactions are designated as one. Thus this incubation time was considered as the " end point." At 15 minutes the majority of the reactions are two and at 1 hour four.

TABLE II.—*Distribution of Graded Gomori Reactions in 46 Transplanted H2712 Mammary Carcinomas.* (*Series II.*)

| Reaction intensities. | 1 min. | 5 min. | 15 min. | 1 hour. |
|---|---|---|---|---|
| 6 | | | | |
| 5 | | | | 4 |
| 4 | | | | 25 |
| 3 | | | 5 | 17 |
| 2 | | 1 | 24 | |
| 1 | 1 | 32 | 17 | |
| 0 | 45 | 13 | | |

The alkaline phosphatase reaction was similar to Series I in so far as the tumor cells and stroma was concerned. The distribution was, however, strikingly different from the other tumors and will be referred to in the subsequent section.

Series III consisted of 48 transplanted dbrB mammary carcinomas. A total of 192 slides were examined, representing the total incubation times of this series. Other organs were also examined in a similar manner. The distribution of the reaction intensities are shown in Table III. At 1 minute incubation time all 48 reactions were negative and at 5 minutes 46 of 48 reactions were negative. The " end point " appeared to be at 15 minutes incubation time as 47 of 48 reactions were of the order of one.

TABLE III.—*Distribution of Graded Gomori Reactions in 48 Transplanted dbrB Mammary Carcinomas.* (*Series III.*)

| Reaction intensities. | 1 min. | 5 min. | 15 min. | 1 hour. |
|---|---|---|---|---|
| 6 | | | | |
| 5 | | | | 12 |
| 4 | | | | 27 |
| 3 | | | | 7 |
| 2 | | | | 2 |
| 1 | | 2 | 47 | |
| 0 | 48 | 46 | 1 | |

Series IV consisted of 48 transplanted 15091a mammary carcinomas as shown in Table IV. A total of 336 slides was examined in this series. There was no reaction present in this series with the incubation times as indicated even up to $2\frac{1}{2}$ hours. The tumor was examined for stroma but very little was present and this showed a negative reaction. The vessels throughout the tumor showed a positive reaction. Other organs were examined in a similar manner.

TABLE IV.—*Distribution of Graded Gomori Reactions in 48 Transplanted 15091a Mammary Carcinomas. (Series IV.)*

| Reaction intensities. | 1 min. | 5 min. | 15 min. | 1 hr. | $1\frac{1}{2}$ h. | 2 hr. | $2\frac{1}{2}$ hr. |
|---|---|---|---|---|---|---|---|
| 6 | | | | | | | |
| 5 | | | | | | | |
| 4 | | | | | | | |
| 3 | | | | | | | |
| 2 | | | | | | | |
| 1 | | | | | | | |
| 0 | 48 | 48 | 48 | 48 | 48 | 48 | 48 |

EXPLANATION OF PLATES.

FIG. 1.—Tumor E0771 transplanted into C57BL and $F_1$ hydribs (Series I). Alkaline phosphatase reaction at 15 minutes' incubation time slides show irregular areas of activity. × 120.

FIG. 2.—Tumor 15091a transplanted into $F_1$ hybrids of C57BL (Series IV). No alkaline phosphatase reaction at 15 minutes' incubation time. × 120.

FIG. 3.—Tumor dbrB transplanted into DBA (Series III). Alkaline phosphatase reaction at 15 minutes' incubation time shows a tendency of the peripheral cells of islands to show more activity than central portion. The supporting connective tissue shows no reaction. × 120.

FIG. 4.—Same tumor and series as Fig. 1, except incubation time is 1 hour. The marked intensity of the alkaline phosphatase reaction obscures detail. × 120.

FIG. 5.—Same tumor and series as that in Fig. 2 except the incubation time is 1 hour. The alkaline phosphatase reaction is still negative at this incubation time. Note the positive reacting vessels in the tumor. × 120.

FIG. 6.—Same tumor and series as in Fig. 3 except incubation time is 1 hour. The alkaline phosphatase activity is most intense in the peripheral cells of the islands. The stroma and vessels are entirely negative. × 120.

FIG. 7.—Tumor H2712 transplanted into C3H (Series II) under high power magnification. Note the peculiar and intense distribution at the borders of cells lining the lumens. In the center is also seen Gomori positive acellular material in a very small lumen. A small globule of positive material seen in a cell in the lower third, right. × 500.

FIG. 8.—A hematoxylin and eosin preparation of Tumor E0771 showing loose reticular stroma and islands of tumor tissue with small lumens. × 120.

FIG. 9.—A hemotoxylin and eosin preparation of Tumor H2712 showing in the central portion a dense stroma. Tumor islands are composed of closely packed cells and acinar structures. × 120.

FIG. 10.—A hemotoxylin and eosin preparation of Tumor dbrB showing the more solid and lobular structure with a loose cellular stroma with many large thin-walled vessels. The cells are smaller than in Fig. 8 and 9 and there is less tendency to lumen formation. Small lumens can be seen in the center of lobules. × 120.

FIG. 11.—A hemotoxylin and eosin preparation of Tumor 15091a showing a dense solid tumor. Cells irregular in size and shape. An occasional lumen is shown in the center. × 120.

Pearson and Richardson.

*Histology and enzyme reaction in the tumor.*

It would be highly desirable if one could relate certain histological and functional features to the enzymatic histochemistry of the tumor. However, in order to make such a comparison a large variety of tumors would have to be studied in inbred strains of mice and with comparable methods by several investigators. It is hoped that the following comparative descriptions might be of some value to future investigators in the clarification of the problems involved.

A visual comparison of the phosphatase activity of three tumors E0771, 15091a and dbrB is shown in Fig. 1 to 6. Fig. 1, 2, 3, show Tumors E0771, 15091a and dbrB respectively at 15 minutes and Fig. 4, 5, 6 at 60 minutes incubation period. It is quite evident that 15091a exhibits no alkaline phosphatase activity and further extension of the incubation time to 2½ hours showed no reaction except in the vessels.

In Series I Tumor E0771 is composed of irregular islands of tumor tissue. These vary in size and shape. They are composed of large cells with large vesicular nuclei which also vary in size and shape. Numerous mitotic figures can be seen throughout. The islands are fairly solid with the exception of small areas where lumens can be seen. There seem to be attempts to form tubular structures. The larger lumens are lined by several rows and layers of cells. Most of the lumens are very small. The interstitial tissue is rather sparse and composed of loose reticular material with many large thin-walled vessels. Fig. 8 shows a photomicrograph of this tumor stained by hemotoxylin and eosin showing the islands of tumor tissue with an attempt at luminal and tubular formation. The interstitial tissue seen to the left is loose and reticular in nature.

The phosphatase reaction began during the first minute of incubation time with a blackening or graying of the cytoplasm. This gave a gray appearance to the slide except in such areas where the blackening is much more pronounced. At 5 minutes this process is more accentuated. The 15 minute incubation time is shown in Fig. 1 and represents a graying and intense irregular blackening in many areas of the slide. An attempt was made to see what cells or portion of the islands that would become intensified more quickly. It seemed that those acini in which there were several layers present revealed the most intense activity. Also areas of cells of those islands adjacent to vascular channels, showed the most intense activity. Very distinct and abrupt deposits of sulphide can be seen in cells undergoing early degeneration. Here the cell membranes are also exaggerated. With the exception of three instances, no very definite pattern of activity could be ascribed to this tumor. Not much could be gained by studying the tumor at 1 hour because much of the detail is obscured by the intense blackening. The interstitial tissue and vessels showed no activity.

Tumor H2712 in Series II was similar histologically to E0771. It differs somewhat in that the interstitial tissue is more dense and organized. It is composed of similar islands of tumor cells. The cells are large and have irregular and vesicular nuclei with numerous mitotic figures. There seems to be more attempt at lumen formation although these are small. There are no double rows of cells as shown in E0771. Large areas are fairly solid and show no evidence of lumen formation. A hemotoxylin and eosin section of the tumor is shown in Fig. 9. Small lumens can be seen in the tumor tissue. With the Gomori reaction as shown in the high power section in Fig. 7, it is seen that the luminal border of the acini give a very intense reaction at 15 minutes' incubation time. Similarly in the center of the picture is seen an attempt at lumen formation in which the activity is high. The end point of the reaction occurs at 5 minutes. At this time enzyme activity can be demonstrated in many areas in the centres of the island. Large irregular clefts can be distinguished and stand out prominently by the activity of the enzyme of the cells lining the lumens. Intraluminal material which is not apparent in the hemotoxylin and eosin preparation shows intense activity. Small globules are present in the cytoplasm of many cells adjacent to lumens or attempted lumen formation. The connective tissue and vessels do not react.

Tumor dbrB in Series III under hemotoxylin and eosin stain shows a more lobular distribution than either E0771 or H2712 and is shown in Fig. 10. The cells and nuclei are smaller than those described for the other series. Numerous mitosis are seen. Lumen formation is sparse but where they are seen they are similar to the other 2 tumors. The interstitial tissue is composed of loose connective tissue, connective tissue and large thin-walled vessels.

With graded Gomori stains the reaction has an " end point " at 15 minutes. Here the areas of activity are irregular thoughout the tumor. Some activity is present in the small lumens but this is not marked. There is a tendency for increased activity at the periphery of the lobule as shown in Fig. 3 and 6. At 1 hour incubation time (Fig. 6) the reaction becomes much more intense. The loose supporting connective tissue and vessels show no activity.

In Series IV Tumor 15091a differs somewhat from the other 3 in that it is a fairly solid tumor not composed of islands or lobules. There are numerous mitosis and the cells vary more in size and shape. The nuclei are for the most part vesicular, very rarely is there evidence of lumen formation. Fig. 11 shows the hemotoxylin and eosin preparation with a small alveolar formation in the center.

With graded Gomori reactions as carried out on the other tumors, this shows a consistently negative reaction in all its incubation times up to $2\frac{1}{2}$ hours. Fig. 2 shows the absence of phosphatase activity at 15 minutes and Fig. 5 at 1 hour. However, in this tumor the vessels show a consistent positive reaction as seen in Fig. 5. Also here is shown in the center, a few small lumens.

*Other Organs..*

Several other organs were studied in a similar manner to that of the tumor. The spleen revealed no change until 15 minutes of incubation and then the reaction began with the blackening of the central vessels of the follicle. At 1 hour this showed rather marked activity. The cells of the peripheral portion of the nodules showed most intense activity. There is no essential difference between the various series.

The liver shows, for the most part, two types of reaction. The commonest begins at the lining of the sinusoids involving the Kupfer cells. The other type of reaction begins at the periportal areas without involvement of the sinusoids. Although the reaction begins for the most part at the 15-minute incubation period it varies considerably as to.extent and localization.

The kidney sections are similar in all series and begins in the brush border of the convoluted tubules. The ovary begins in most series with the 5 minute incubation time in the theca externa which shows progressive activity to 1 hour. Small stromal vessels also show activity. All the series were approximately the same. The reaction in the testes began at 5 minutes and became more intense at

1 hour incubation. For the most part it only involved the capsule surrounding the spermatic epithelium. Very little reaction was present in the adrenals and occurred for the most part at 1 hour incubation time where the reaction was present in the vessels in the glomerular zone.

### DISCUSSION.

By means of graded Gomori reactions a more complete picture could be obtained concerning differences between 4 tumors arising in the breast tissue of mice. These tumors have been transplanted for many generations to their original hosts and $F_1$ hybrids with a 100 per cent take. Thus a certain homogeneity of the tumor strain should be expected. The three tumors E0771, H2712 and dbrB are fairly identical in histology, as each is an adenocarcinoma with attempts at acinar formations. This latter feature is slightly more developed in E0771 and H2712 than in the dbrB. One difference that occurs in the E0771 is the presence of double layers of tumor cells in the attempt at acinar formation. In the 15091a the tumor is solid with very few small acini.

The overall phosphatase reaction was very intense in E0771, very much less in H2712 and bdrB and none in 15091a. This was evident in spite of the fact that the rate of growth of the tumor was about the same in all the series. Numerous mitoses were present in all the tumours. Hard, Pratt-Thomas and Belkin (1948) felt that there was a loss of alkaline phosphatase activity in the more anaplastic tumor cells. Although it would seem in our series that the anaplastic Tumor 15091a having no reaction would point toward such contention, the E0771 is a very rapidly growing tumor with numerous mitoses. We would prefer to delay our judgment on this issue until a larger variety and strains of tumors have been studied.

An interesting and significant paper which has come to our attention as this work was completed was the observation of Shelton (1952). This investigator found a tumor in a 6-month-old Strain A female mouse that had been exposed to 400 r whole body X-radiation on the date of birth. Lymphoma 1 was propagated in A mice from a large tumor mass located in the anterior mediastinum. Lymphoma 2 was propagated by subcutaneous injection into Strain A mice of fragments of lymph nodes from a mouse bearing the original tumor. Strain 1 grew as a localized tumor with local invasion but no metastasis, but Strain 2 reacted as an acute leukemia with rapid growth infiltration of the organs and high peripheral count.

In determination of the alkaline phosphatase activity it was found the Lymphoma 1 showed no reaction while Lymphoma 2 showed a reaction as expressed by 5 mg. phosphorus per milligram of tissue per hour. No detectable reaction could be seen by the Gomori method in Lymphoma 1 whereas Lymphoma 2 was strongly positive. Here we have a rapidly growing persumably anaplastic tumor showing an increase in alkaline phosphatase reaction.

In our laboratory we have also determined the alkaline phosphatase activity as expressed in gamma of phosphorus per milligram of nitrogen at 30 minutes incubation time in a large series of these 4 mammary tumours. We have found that the specific activity of alkaline phosphatase thus expressed in gamma of phosphorus was 701 $\pm$ 30 in the E0771 tumor, 157 $\pm$ 3·4 in the dbrB tumor, 30 $\pm$ 4·0 in the H2712 and only 5 $\pm$ 0·6 in the 15091a tumor. This corresponds to the histochemical findings of a very high and low values in E0771 and 15091a respectively. There is a discrepancy in the H2712 tumour which shows an " end point " at 5 minutes in contrast to dbrB which shows it in 15 minutes. That this discrepancy is probably due to differences in the localization of enzyme and incubation times, thus there is a sharp localization of enzyme activity in the luminal border of the cells of H2712 which is not the case in dbrB. These tumours were transplanted into the same hosts and under the same conditions as the four series reported here. In the chemical reaction the incubation times were taken only at 30 minutes whereas we were concerned with several incubation times in our histochemical reactions.

The distinctive localization of Tumor H2712 is of interest in so far as its histological appearance is quite similar to all except 15091a. The fact that the luminal borders of the cells as well as accumulation into the lumen of Gomori positive material which is acellular suggests a secretory process which has been retained by this tumor.

### CONCLUSIONS.

1. Four series of transplantable tumors arising from the breast in mice were transplanted to their original hosts and $F_1$ hybrids.
2. Enzyme activity as expressed by graded Gomori reactions with 4 incubation times indicated overall differences in alkaline phosphatase activity from a very intense reaction to no reaction.
3. Variation in localization of the reaction in one tumor (H2712) was striking as there was accumulation of activity in the luminal border and activity within lumens suggesting a secretory process.
4. Very little difference histologically could be seen in 3 tumors (E0771, H2712, and dbrB) to account for variations in overall intensity as well as such localization difference that existed.
5. The biochemical data seem to correlate very closely to the histochemical data, especially where wide differences exist.

We wish to express our appreciation to Mr. Frank Mallory and Mr. Rodney Galbraith for photographic work done on this paper.

This investigation was supported by a research grant from the National Cancer Institute, U.S. Department of Health, Education and Welfare.

### REFERENCES.

Carter, T. C., Dunn, L. C., Falconer, D. S., Grüneberg, H., Heston, W. E., and Snell, G. D.—(1952) *Cancer Res.*, **12**, 602.
Cloudman, A. M.—(1928) *Amer. J. Cancer*, **16**, 568.
Greenstein, J. P.—(1947) 'The Biochemistry of Cancer,' p. 305. 1st edition. New York (Academic Press, Inc.).
*Idem.*—(1945) 'Enzymes in Normal and Neoplastic Animal Tissues,' pp. 192–222. 1st edition. Washington (*Amer. Ass. Adv. Sci.*).—(1942) *J. nat. Cancer Inst.*, **2**, 511.
Hard, W. L., Pratt-Thomas, H. R., and Belkin, M.—(1948) *S. Dak. J. Med. Pharm.*, **1**, 154.
Kabat, E. A., and Furth, J.—(1941) *Amer. J. Path.*, **17**, 303.
Little, C. C., and Strong, L. C.—(1924) *J. exp. Zool.*, **41**, 92.
Pearson, B., and Morrione, T.—(1949) *Cancer Res.*, **9**, 564.
*Idem*, Novikoff, A., and Morrione, T.—(1950) *Ibid.*, **10**, 557.
Shelton, Emma.—(1952) *J. nat. Cancer Inst.*, **12**, 1209.
Snell, G. D., Cloudman, A. M., and Woodworth, E.—(1948) *Cancer Res.*, **8**, 429.
Strong, L. C., and Little, C. C.—(1920) *Proc. Soc. exp. Biol. N.Y.*, **18**, 45.